# Reem-Shape Phononic Crystal for Q Anchor Enhancement of Thin-Film-Piezoelectric-on-Si MEMS Resonator

**DOI:** 10.3390/mi14081540

**Published:** 2023-07-31

**Authors:** Mohammed Awad, Temesgen Bailie Workie, Jing-Fu Bao, Ken-ya Hashimoto

**Affiliations:** School of Integrated Circuits Science and Engineering, University of Electronic Science and Technology of China, Chengdu 611731, China; wtbailie@std.uestc.edu.cn (T.B.W.); k.hashimoto@ieee.org (K.-y.H.)

**Keywords:** MEMS resonators, TPoS, phononic crystals, quality factor, anchor loss

## Abstract

This paper proposes a reem-shaped phononic crystal for the performance enhancement of TPoS resonators. The proposed phononic crystal offers an ultra-wide acoustic band gap that prevents energy leakage through the supporting substrate upon its placement at the anchoring boundary, resulting in significant improvements in the resonator quality factor. Simulated results show reem-shape phononic crystals generate a band gap up to 175 MHz with a BG of 90% and enhance the anchor quality factor from 180,000 to 6,000,000 and the unloaded quality factor from 133,000 to 160,000, representing 33.3-fold and 1.2-fold improvements, respectively.

## 1. Introduction

Micromachined resonators have a great possibility for integration with microelectronics at the die or package level [1,2,3,4,5]. This advantage leads to reduced cost, a smaller form factor, enhanced performance, and decreased fabrication complexity. Micromachined resonators are grouped into three main categories: piezoelectric resonators, capacitive resonators, and piezoresistive resonators. Piezoelectric micromachined resonators have proven excellent performance and strong reliability in timing applications and also have good merits such as a strong electromechanical coupling coefficient [6], a large Q, better temperature stability, and the possibility of the resonator being implemented and fabricated on a silicon substrate at the die level [7].

Micromachined resonators with large Q are commonly used in oscillators with low phase noise and filters with low insertion loss [8]. However, before MEMS resonators spread commercially in markets over other types of resonant devices, some loss factors should be mitigated. These losses are thermo-elastic damping (TED), support (anchor loss) [9,10], material loss [11], and other losses like resistive loss, surface loss, and dielectric loss [12,13]. In this regard, the quality factor of the micromachined resonator consists of many branches, such as anchor quality factor (Qanchor), electrode quality factor (Qelectrode), TED quality factor (QTED), material quality factor (Qmaterial), and unknown quality factor (Qunknown). The equivalent Q can be expressed with the following equation [14]:

(1) $$Q=2\pi \;\frac{E_{stored}}{E_{loss}}$$

where *E_stored_* and *E_loss_* represent the stored and dissipated energy in the resonator, respectively. Anchor loss represents the major loss of the piezoelectric resonators vibrating in width extension mode. This loss is due to the radiation of acoustic waves to the supporting substrate through tethers. One of the mechanisms used to mitigate this loss is using acoustic reflectors [15,16]. The shortcoming of this method is its inefficiency in reducing anchor loss. The other recently introduced method of improving the *Q* of the resonator is using energy-preserving suspended frames [17]. Even though it is reported to provide a significant improvement in *Q*, it introduces spurious modes near the intended resonance mode. The most widely used method is to apply one- or two-dimensional phononic crystals on anchors of the resonator or on supporting tethers, which is provided by many researchers [18,19,20]. Different types of effective phononic crystals have been achieved. This article introduces a new 2-D phononic crystal structure (Reem-PnC) that generates a wide acoustic band gap up to 175 MHz, resulting in a high anchor quality factor of 6,000,000.

## 2. Phononic Crystal & Theory of Wave Propagation

### 2.1. Principle of Wave Propagation in PnCs

The periodic structure of phononic crystals consists of two or more elastic materials with excellent mechanical properties [21,22,23]. Properly designed phononic crystals provide some range of frequencies in which acoustic waves are inhibited from propagating. This range of frequencies is defined as an “acoustic band gap” [24]. Bloch’s theorem is usually used to characterize the propagation of waves in acoustic mediums (phononic crystals) and periodic dielectric mediums (photonic crystals) [25]. The equation of the acoustic wave propagation in material with anisotropic nature can be written as [26,27,28]:

(2) $$\frac{\partial }{\partial x_{j}}\left(C_{ijkl}\frac{\partial u_{k}}{\partial x_{l}}\right)=\rho {ϋ}_{i}$$

where *x_j_* represents the coordinate axes (*x, y, z*), *C_ijkl_* represents the tensor of elastic material, *u_i_* represents the components of displacement (*u_x_, u_y_, u_z_*), and ρ is the silicon density. To calculate the bandgap, the two boundary destinations on the unit cell (a) are adjusted to Bloch’s periodic boundary conditions through all the propagation directions. The Bloch-Floquet theorem verifies the periodic boundary condition of displacements as defined by [23]:

*u*_*i*_* (x,y,z + a,t) = u*_i_ *(x,y,z,t) e*^*jka*^ (3)

where *k* and a represent the wave number and the lattice constants of the PnC. All frequency eigenmodes can be calculated by sweeping *k* through the boundaries of the first irreducible Brillouin zone (IBZ) in the single structure of the phononic crystal. The frequencies are a function of *k* (*k* = *ω*/*c*), where c represents acoustic wave velocity along the (110) direction of silicon and ω represents angular frequency. The relation among generated displacement due to stress can be expressed as [23]:

*u*_*i*_*(x + a,t) = e*^*jk.a*^ *u*_*i*_*(x, t)* (4)

*σ*_*ij*_*(x, + a,t) = e*^*jk.a*^ *σ*_*ij*_*(x, t)* (5)

The Bloch profile shows the calculations of changes in displacements, eigenfrequencies, and stress fields as *k* varies gradually. Many curves are achieved between *k* and *ω* due to varying wave vectors through all highly symmetric edges of the first IBZ of the Reem-PnC. All the calculations of equations and solutions are done using COMSOL Multiphysics through the FE analysis method.

### 2.2. PnC Design

**Figure 1:**
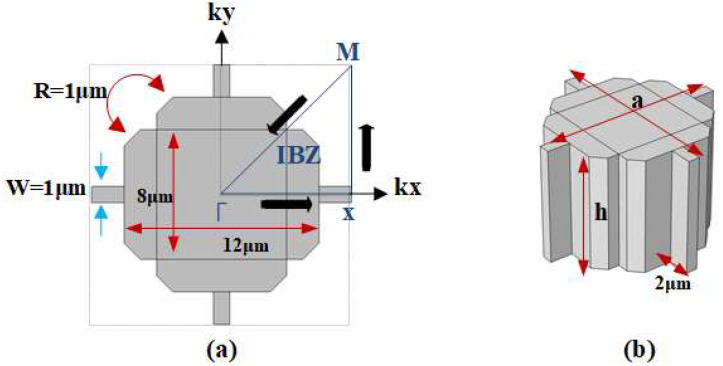
(**a**) 2-D view of Reem-PnC with the first irreducible Brillouin zone (IBZ) (**b**) 3-D view of Reem-PnC.

The Reem-PnC is illustrated in Figure 1a,b. The structure of a single unit cell with lattice constant a = 16 μm consists of two cross blocks perpendicular to each other with widths and lengths of 12 μm, 8 μm, and 8 μm, 12 μm, respectively, a high of h = 10 μm, and chamfered edges of radius R = 1 μm. This PnC is connected to arrays of PnCs by four connectors with widths and lengths of 2 μm and 1 μm. All the designs were constructed using single-crystal silicon, the mass density of silicon ρ = 2330 kg/m^3^, and the elastic constants illustrated in Table 1.

**Figure 2:**
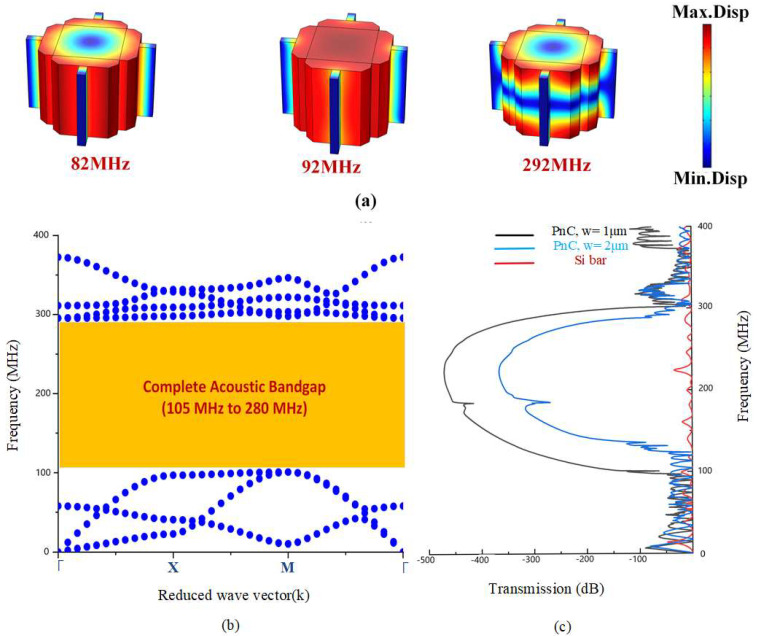
Illustration of: (**a**) the first eigenfrequency mode shape in the band structure of Reem-PnC, (**b**) band structure through (Γ-X-M-Γ) direction of the IBZ of Reem-PnC and (**c**) transmission of S21 parameters of an array of Reem-PnC unit cells.

The axes (*x, y,* and *z*) of the lattice synchronized with the (110), (110), and (001) directions of the original orientation of the silicon wafer (i.e., 100). Adding Floquet periodic boundary conditions at the edges of the unit cell and sweeping the parameters of k through the direction of Γ-X-M-Γ for the first IBZ, as illustrated in Figure 1a. The first eigenfrequency modes of Reem-Shape PnC with the associated bandgap are illustrated in Figure 2a,b. The desired design of PnC achieves a complete bandgap of 175 MHz in the frequency range between 105–280 MHz with a (w) of 1 μm and 135 MHz in the frequency range between 125–275 MHz with a (w) of 2 μm.

Reem phononic crystal generates a wide bandgap from 105 MHz to 280 MHz. The ratio between gap and mid-gap is determined by the equation [24]:

(6) $$Bandgap=\left(\frac{f_{top}-f_{bot}}{\frac{f_{top}+f_{bot}}{2}}\right)$$

where *f_bot_* and *f*_top_ represent the open and closed frequencies of the bandgap. A wide acoustic bandgap is obtained with a BG of 90% from Reem-PnC dimensions as mentioned above with a connector of w = 1 μm; changing the connector width to 2 μm achieves a decrease in bandgap BG of 75% as shown in Figure 2c. The comparison of Reem-PnC with other PnC shapes is illustrated in Table 2. The filling fraction of the Reem-PnC, which represents the area of PnC relative to the area of the lattice, is equal to 0.515, and it is calculated using the equation [24]:

(7) $$filling\;fraction=\frac{area\;of\;PnC}{area\;of\;lattice}$$

## 3. Transmission Characteristics of Reem-PnC

**Figure 3:**
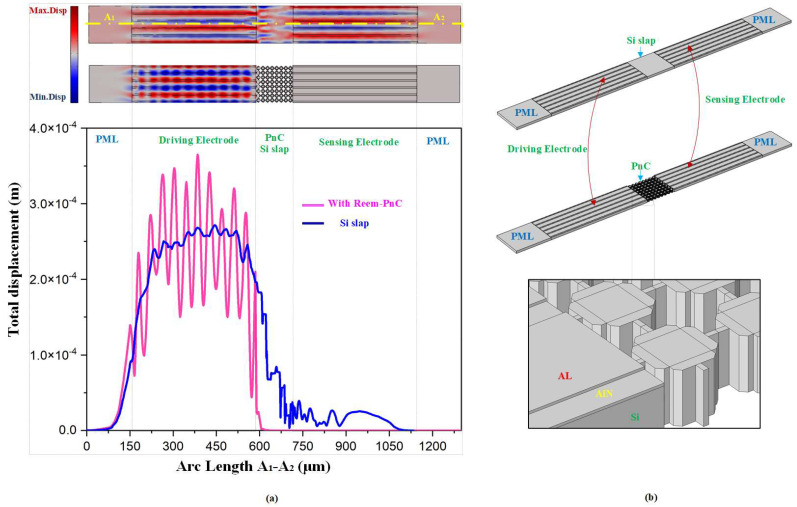
(**a**) Displacement distribution of the transmission medium with solid Silicon plate delay line and Reem-PnC array at 191 MHz on the A1–A2 line (**b**) 3D view of sense and drive electrodes with silicon plate and Reem-PnC.

To prove the formation of the acoustic bandgap by the Reem-PnC structure, the transmission characteristic is analyzed using two acoustic delay lines. As shown in Figure 3b, the Reem PnC plate and silicon plate (two different transmission mediums among sense and drive electrodes) are applied to realize the transmission characteristics of the desired PnC. The end of each delay line boundary in the x-direction is perfectly matched to effectively decrease the reflected wave interface. The drive electrode is excited by 0.01 watts, and the sense electrodes are terminated at 0.0 watts. The transmission (S21) is calculated using the following relationship [29]:

(8) $$S21\left(dB\right)=10\;log_{10}\;\left(\frac{P_{out}}{P_{in}}\right)$$

where *P_in_* and *P_out_* are the input and output power transferred to the Reem-PnC delay line and solid silicon delay line, respectively, as shown in Figure 3b. S21 represents the transmission power coefficient between the input and output ports. Clearly from Figure 3b, the S21 transmission spectrum with an array of Reem-PnC proofs the proposed design successfully forms an acoustic bandgap. The finite element simulation results (displacement profile) illustrate that the wave is strongly attenuated in the transmission spectra at the beginning of the Reem-PnC delay line and continues to zero, compared to the silicon plate delay line. Reem-PnC satisfies that there is a strong prohibition on the propagation of acoustic waves.

## 4. Resonator Design

**Figure 4:**
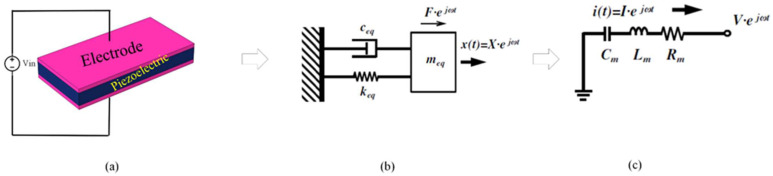
(**a**) Illustration of piezoelectric excitation in MEMS resonator (**b**) equivalent mechanical system of the resonator (**c**) equivalent electrical circuit of the resonator.

The resonator design can be realized as a mass-spring-damper system. All vibrating systems have energy dissipation mechanisms. This mechanism is characterized simply as a damper. The union of the mass-spring-damper system is the basic model for the resonator. Clearly, from Figure 4b, Newton’s second law of motion realizes the relations between the motion of mass and input force, which are generally expressed by [1]:

(9) $$m_{eq}\frac{\partial^{2}x}{\partial t^{2}}+c_{eq}\frac{\partial x}{\partial t}+k_{eq}x=F\;\;\;\;\;\;\;\;\;\;\;\;\;\;\;\;\;\;\;\;\;\;\;$$

where *m_eq_* represents equivalent mass, *F* represents applied force, *k_eq_* represents equivalent stiffness, and *c_eq_* is the equivalent total loss. The relationship between the input and output of the system can be expressed as [1]:

(10) $$\;\;\;\;\;\;\;\;\;\;\;\;\;\;\;\;H\left(s\right)=\frac{X\left(s\right)}{F\left(s\right)}=\frac{1}{m_{eq}^{2}+c_{eq}s+k_{eq}}=\frac{1}{k_{eq}}\left(\frac{\omega_{n}^{2}}{s^{2}+\omega_{n}Q^{-1}S+\omega_{n}^{2}}\right)$$

where (*s*) is defined as complex frequency, *Q* is defined as body quality factor, and $\omega_{n}$ defined as the natural frequency. The resonant frequency for systems of second order is given by [24]:

(11) $$\omega_{n}=2\pi f_{n}=\sqrt{\frac{k_{eq}}{m_{eq}}}\;\;\;\;\;\;\;\;\;\;\;\;\;\;\;\;\;\;\;\;\;\;\;\;\;\;\;\;\;\;\;\;\;\;\;\;\;\;$$

The relation between natural frequency *ω_n_* and the resonance frequency *ω_r_* of the system of second order is expressed by [1]:

(12) $$\;\;\;\;\;\omega_{r}=\omega_{n}\sqrt{1-\frac{1}{2Q^{2}}}$$

It is clear from the above relation that for high *Q*, as in MEMS resonators, the natural frequency equals the resonance frequency (i.e., *ω_r_* ≈ *ω_n_*). By applying a single frequency analysis, the resonance frequency of the MEMS resonator can be extracted from the displacement curve in the frequency response by using the general formula [20]:

(13) $$Q=\frac{f_{r}}{\Delta f\left(-3dB\right)}$$

where Δ*f*(−3*dB*) is defined as the −3 dB bandwidth between the resonant frequency and the frequency response curve. In all cases, the resonator is expressed as an electric circuit with series R, L, and C. Figure 4b,c show an equivalent mechanical and electrical model for a MEMS resonator. The input voltage represents the input force, the current represents velocity, the damping loss is represented by *R_m_* (motional resistance), the *C_m_* (motional capacitance) is represented by the inverse of stiffness, and the mass is represented by the motional inductance *L_m_*. The relation among *R_m_, C_m_, Q_u_* (unloaded quality factor), and insertion loss IL of the resonator is given by [3]:

(14) $$R_{m}=\frac{1}{Rel\left(Y_{11}\right)},\;\;\;\;R_{m}=\frac{1}{2\pi f_{r}C_{m}Q_{u}}$$

where Y_11_ is the admittance curve of the resonator.

**Figure 5:**
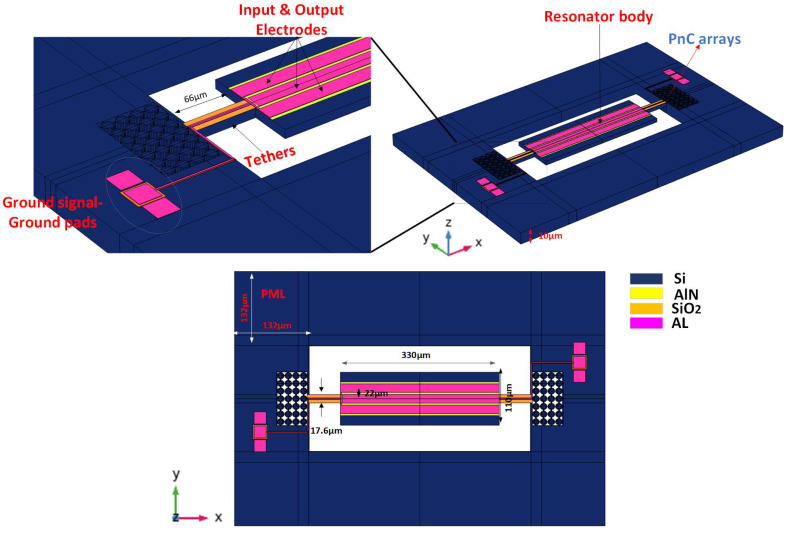
A 3-D view of the resonator with arrays of Reem-PnC.

A 5th-order piezoelectric on silicon MEMS resonator was implemented, simulated, and analyzed using COMSOL Multiphysics, as demonstrated in Figure 5. A thin film of the AlN layer is bonded to a silicon substrate. The Al electrode positioned on top of piezoelectric material with a depth of 0.5 μm excites the vibration on the piezoelectric resonator by applying a voltage of 1 V, while the gap between the two electrodes is set to be 0.4 μm. The dimensions of the resonator are 110 μm width and 330 μm length, respectively. The wavelength of the resonator λ is equal to 44 μm. The tether length and width of the desired resonator are set to 1.5 λ and 17.6, respectively. A Reem-PnC consists of two cross blocks perpendicular to each other with width and length of 12 μm and 8 μm, respectively, chamfering radius R at the edges of 1 μm, and lattice constant a = 16 μm. The PnC arrays are positioned externally in anchors to generate a bandgap that coincides with the resonant frequency to prohibit acoustic wave propagation to the device substrate and cause a loss in energy. The resonant frequency *(f_r_)* of the resonator body can be obtained from [16]:

(15) $$f_{r}=\frac{1}{\lambda }\sqrt{\frac{E}{\rho }}$$

where *λ* defined as wavelength, E is defined as Young’s modulus in (110) axes, and *ρ* defined as the density of single-crystal silicon. The *f_r_* calculated from Equation (16) is around 191 MHz in the desired design *λ* = 44 μm. The resonator design parameters are listed in Table 3.

## 5. Techniques for Anchor Loss Enhancement in the Resonator

**Figure 6:**
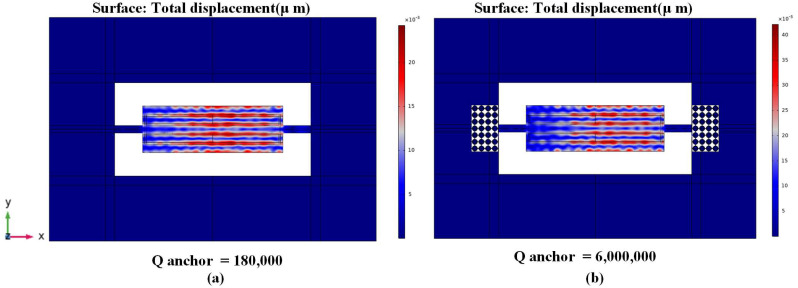
5th-order eigen mode shape and the calculated *Q_anchor_* (**a**) without PnC and (**b**) with Reem-PnC.

A 2-dimensional array of Reem-PnC is deployed in the anchoring boundaries of the resonator to enhance the anchor quality factor and, as a result, the total quality factor (*Q_tot_*). From Figure 6, the *Q_anchor_* of the TPoS MEMS resonator can be generally obtained from the resonance frequency divided by the −3 dB of bandwidth of the resonance maximum point in displacement profile at the frequency response [16]:

(16) $$Q_{anchor}=\frac{f_{r}}{\Delta f\left(-3dB\right)}$$

where *f*_r_ is defined as resonance frequency, the resonator mode shape with and without PnC and associated anchor quality factor are shown in Figure 6.

## 6. Calculation of Resonator Performance

Simulated S21 parameters and admittance Y11 curves of a desired resonator with and without Reem-PnC were calculated by the FEA simulation at the frequency domain in COMSOL Multiphysics to obtain the insertion loss, loaded Q, unloaded Q, the figure of merit, effective electromechanical coupling coefficient (k^2^_eff_), and motional resistance R_m_. The relationships of *Q, Q_u_, FoM*, and *k^2^_eff_* are given by [30]:

(17) $$Q_{l}=\frac{f_{r}}{\Delta f_{\left(-3dB\right)}},\;Q_{u}=\frac{Q_{l}}{1-10^{\left(\frac{-IL}{20}\right)}},\;k_{eff}^{2}=\frac{\pi^{2}}{8}\left(\frac{f_{s}^{2}-f_{p}^{2}\;}{f_{p}^{2}}\right),\;FoM=Q_{u}*k_{eff}^{2}$$

## 7. Discussion

**Figure 7:**
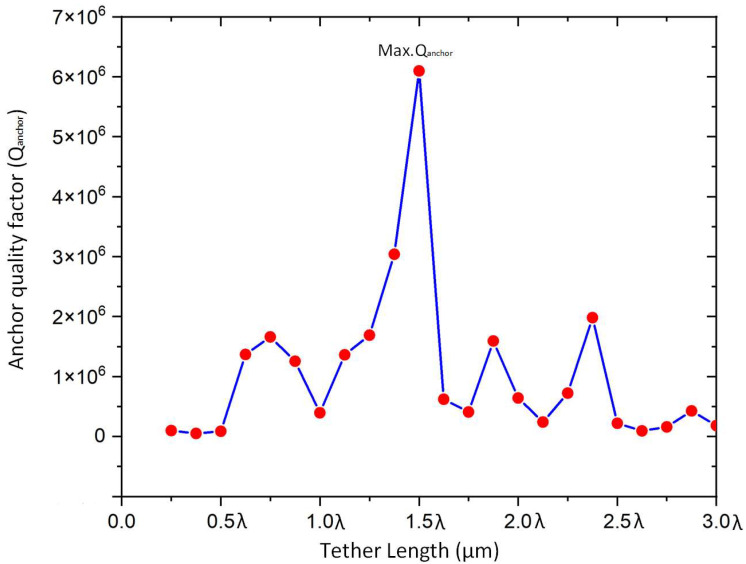
Illustration of the relation between different values quality factor and tether length according to wavelength.

Figure 7 presents different values of the anchor quality factor as a function of longitudinal wavelength (λ. The minimum value of the anchor quality factor exists at the tether length equal to integer multiples of the wavelength (i.e., 1*λ, 2*λ, 3*λ), while the maximum points of Q are obtained at the integer multiples of a quarter of the wavelength. The maximum *Q_anchor_* was achieved at 1.5 λ.

**Figure 8:**
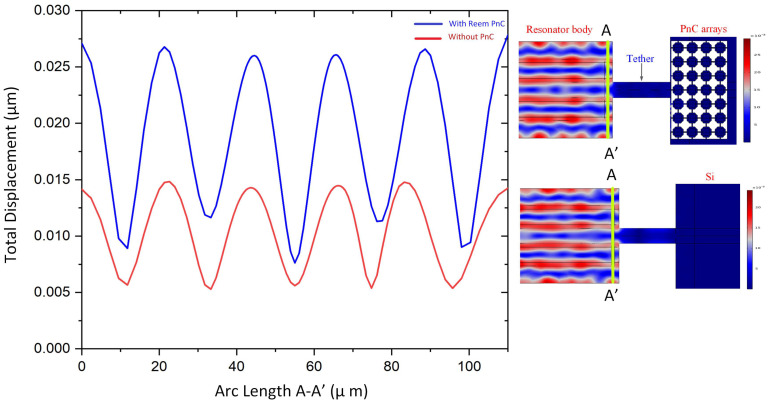
Illustration of: A–A′ line total displacement (μm) of the resonator with and without Reem-PnC.

Figure 8 illustrates the displacement plot across the line A-A′, which verifies the proposed resonator with Reem-PnC and offers a high enhancement in the total displacement of the resonator in comparison with the resonator without PnC. This verifies that the amount of stored energy in the resonator with Reem-PnC is larger than the resonator without PnC. 

**Figure 9:**
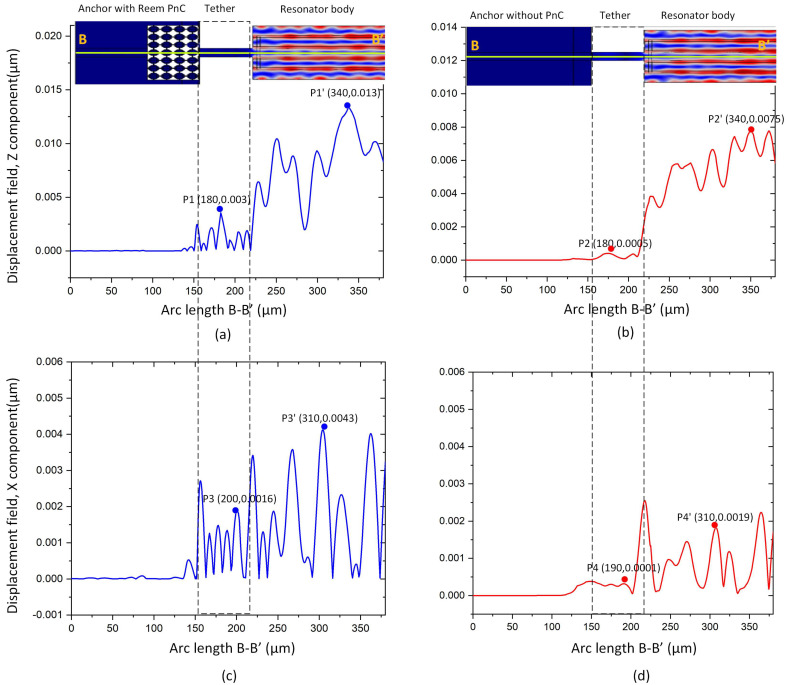
Illustration of: B–B′ displacement (μm) z component of the resonator (**a**) with and (**b**) without Reem-PnC, B–B′ displacement (μm) x component of the resonator (**c**) with and (**d**) without Reem-PnC.

Figure 9a,b illustrates the absolute value of the *z* component displacement field along the B–B′ line in the two resonators with and without Reem-PnC. It is clear from the plot that the displacement at peak point P1 in the tether is higher than the displacement at peak point P2. Continuously, the displacement in the resonator is enhanced in comparison between points P′1 and P′2. Figure 9c,d illustrate the absolute value of the *x* component displacement field along the B–B′ line. It is clear from the plots that the displacement of the peak point P3 in the tethers is greater than the displacement in point P4, and the displacement component in the resonator is enhanced in comparison between points P′3 and P′4. The displacement components *x* and *z* in the resonator with Reem-PnC are greater than those in the resonator without PnC.

**Figure 10:**
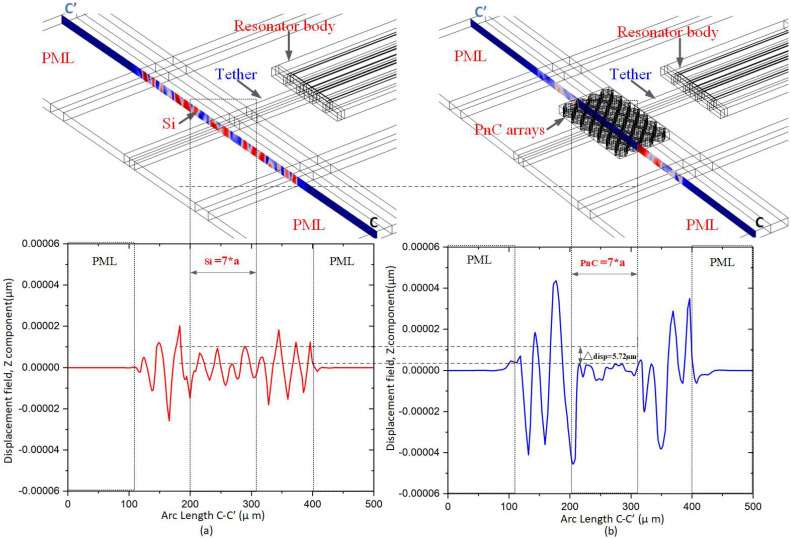
Illustration of: B–B′ displacement (μm) z component of the resonator (**a**) with and (**b**) without Reem-shape PnC.

It is clearly shown from Figure 10 that there is no significant displacement in the PnC array at the anchor boundary of the resonator with Reem-PnC as in cutting plane C-C′. Also, the displacement profile in Figure 10a,b shows there is a displacement increase on the anchoring boundaries of the resonator without PnC (i.e., Δdisp = 5.72 μm). This proves there is energy loss due to energy transverse from the resonant body to the anchor’s boundary.

**Figure 11:**
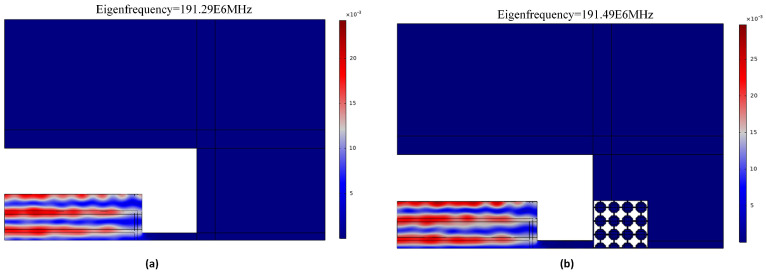
The quarter of 5th order resonator (**a**) without PnC and (**b**) with Reem-PnC.

**Figure 12:**
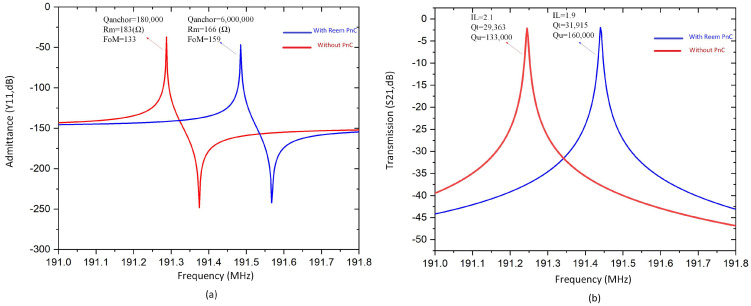
(**a**,**b**) The admittance Y11 and transmission parameter of resonator S21 with and without Reem-PnC.

As is seen from Table 4, the simulated Q_anchor_ and Q_u_ of the designed resonator with Reem-shape PnC are enhanced by 13.5 and 1.2 folds, respectively, in comparison with the resonator without PnC. The remaining parameters of the resonator, such as insertion loss (IL) and motional resistance (Rm), were obtained from the transmission magnitudes (S21, dB) and admittance (Y11, dB) at the quarter of the resonator, as shown in Figure 11. The plots of the parameters are illustrated in Figure 12a,b, respectively. These parameters clearly show enhancement due to the proposed design, while *k_eff_* remains unchanged. 

## 8. Conclusions

This work proposes Reem-PnC as a new phononic crystal design used for anchor loss reduction in thin-film piezoelectric-on-silicon MEMS resonators. The proposed design generates a wide bandgap that prevents acoustic wave propagation to the support structure. Moreover, tether length and width are tuned to optimum dimensions to achieve a high anchor quality factor. The combination of the two approaches achieves a high-quality factor. In this regard, an anchor quality factor of about 6,000,000 and an unloaded quality factor (Qu) of about 160,000 are obtained from the resonator with Reem-PnC, which accounts for 33.3-fold and 1.2-fold enhancement in comparison with the resonator without PnC, respectively.

## Figures and Tables

**Table 1:** Elastic constants of single crystal silicon.

Parameter	Ex (GPa)	Ey (GPa)	Ez (GPa)	σ_yz_	σ_zx_	σ_xy_	Gyz (GPa)	Gzx (GPa)	Gxy (GPa)
Value	169	169	130	0.36	0.28	0.064	79.6	79.6	50.9

**Table 2:** Different PnC shapes in simulated acoustic bandgaps with a resonance frequency in a close range.

PnC Shape	Resonance Frequency (MHz)	Lattice Constant (μm)	Bandgap	BG%
Cross-Shape [20]	138	20	90 to 220	83
Spider Web-like [2]	76	24	68 to 84.5	20.9
Solid disk [21]	134	22	93 to 175	61
Reem-PnC (this work)	191	16	105 to 280	90

**Table 3:** Resonator design parameters.

Parameter	Value (μm)
Resonator length, l	330
Resonator width, W	110
Piezoelectric thickness, Pt	0.5
Electrode thickness, Ew	0.5
Tether length, Tl	1.5λ
Electrode gap	0.4
Resonant frequency, fr	191.49 (MHz)
Wavelength, λ	44
Electrode gap	4
Silicon substrate high	10
Perfect matched layer width	3λ

**Table 4:** Performance of the resonators with Reem-PnC and without PnC.

Resonator	fr (MHz)	Qanchor	IL (dB)	Ql	Qu	K2eff%	Rm (Ω)	FoM
With Reem PnC	191.49	6,00,000	1.9	31,915	160,000	0.10	166	159
Without PnC	191.29	180,000	2.1	29,363	133,000	0.10	183	133
